# Rush Medical College

**Published:** 1853-09

**Authors:** N. S. Davis

**Affiliations:** Sec’ty of the Faculty; Chicago


*Rush Medical College.*

Some little time since it was announced in some of the daily papers of this city that Prof. D. Brainard, President of the Faculty of Rush Medical College, and Professor of Surgery, would soon leave for Europe to spend the ensuing winter. To prevent any misunderstanding on the part of the friends of the College, I am authorized to state that the Faculty have unanimously consented to permit Prof. Brainard to visit Europe during the present season. He goes out expressly for the purpose of procuring such books, apparatus, preparations, and means of illustration, as the interests of the College require, in view of the establishment of a good museum and library in connection therewith. In view of his *temporary* absence, the course of lectures on *Surgical Anatomy*, *Surgery*, and *Clinical Surgery*, will be given by Prof. Herrick. The course on *Descriptive Anatomy* will be given by Dr. J. W. Freer, for several years past Demonstrator of Anatomy in this College. The course on *Physiology* and *Histology*, will be given by Dr. H. A. Johnson, of this city, whose enthusiastic devotion to those branches, and familiarity with the use of the microscope, eminently qualifies him for such a task. All the other branches will remain as in former years, and as specified in the Annual Announcement. Though all will regret even the temporary absence of Prof. Brainard from the post he has so long occupied with distinguished honor, yet, in view of the well-known reputation of Prof Herrick, as a surgeon, both at home and in connection with the army in Mexico, and his long experience as Professor of Anatomy, we feel no hesitation in assuring those interested in the coming session, that the several courses of instruction will be as full and complete in every particular, as during any former College term.

N. S. DAVIS,

*Sec'ty of the Faculty.*

Chicago, Sept., 1853.

